# 2301 Mucoidal pseudomonas aeruginosa infection is associated with regional inflammation in the cystic fibrosis lung

**DOI:** 10.1017/cts.2018.97

**Published:** 2018-11-21

**Authors:** Sankalp Malhotra, Daniel J. Wozniak, Don Hayes

**Affiliations:** 1 Department of Microbial Infection and Immunity, Department of Microbiology, The Ohio State University College of Medicine; 2 Pulmonary Medicine, Transplant Program Nationwide Children’s Hospital

## Abstract

OBJECTIVES/SPECIFIC AIMS: Cystic fibrosis (CF) is a life-shortening genetic disease that affects approximately 30,000 patients in the United States. CF patients suffer from chronic pulmonary infections that are associated with hyperinflammation and irreversible damage to the lower airways. As CF patients age, Pseudomonas aeruginosa (P.a.) is the predominant pathogen that infects the respiratory tract. The P.a. strains initially infecting the CF lung have a nonmucoid colony morphology, whereas, once chronic infection is established, these bacteria mutate leading to the emergence of mucoid P.a. variants with heightened resistance to both antibiotics and host immunity. Both nonmucoid and mucoid P.a. variants are often co-isolated on microbiological cultures of sputum collected from CF patients. However, the CF lung is known to exhibit heterogeneity in inflammation and infecting microbes across different lung regions that cannot be studied using routine sputum collection alone. Here, using a standardized bronchoscopic protocol, bronchoalveolar lavage (BAL) fluid was prospectively collected from each lobe of a CF cohort undergoing clinically indicated surgical procedures. We sought to investigate if there is an association between infecting P.a. variants (nonmucoid, mucoid, or mixed populations), the lung lobes in which these variants are found, and regional proinflammatory cytokine production. METHODS/STUDY POPULATION: We performed BAL on 16 CF patients with clinically stable disease. For each patient, we obtained BAL fluid from the right upper lobe, right middle lobe, right lower lobe, left upper lobe, lingula, and left lower lobe. We plated BAL fluid on nonselective and P.a.-selective medium to quantitate bacteria and to identify P.a. colony subtypes (nonmucoid, mucoid, or mixed). We further used a V-PLEX human cytokine array to quantitate inflammatory cytokine concentrations (IL-1β, TNF-α, IL-6, IL-8, and IL-10) within BAL fluid specimens. Our specimen collection was approved by the local IRB with informed consent and assent obtained from patient volunteers. RESULTS/ANTICIPATED RESULTS: Based on microbiological analysis, each lobar BAL specimen was classified as uninfected with P. a. or infected with nonmucoid, mucoid, or mixed (both nonmucoid and mucoid) P.a. variants. There was no observed propensity of mucoid or nonmucoid variants to be confined to certain lung lobes in our cohort. However, infection with mucoid P.a. variants was associated with higher concentrations of IL-1β (*p*<0.001), TNF-α (*p*<0.001), IL-8 (*p*<0.001), and IL-10 (*p*<0.001) within lobar BAL fluid compared with P.a.-free specimens. Specimens with mucoid variants also had greater concentrations of TNF-α (*p*<0.01), IL-8 (*p*<0.001), and IL-10 (*p*<0.05) compared with specimens with only nonmucoid P.a. variants. Patients infected with mixed mucoid and nonmucoid variants showed higher concentrations of TNF-α and IL-10 (*p*<0.05) as well as nonsignificant trends for higher concentrations of IL-1β and IL-6 compared to P.a.-free samples. Interestingly, the presence of nonmucoid P.a. variants was inversely correlated with IL-6 (*p*<0.05). Total bacterial burden (both P.a. and non-P.a. species) within BAL fluids was positively correlated with higher proinflammatory cytokine concentrations. Additionally, independent of bacterial colonization, the upper lobes (right upper lobe and left upper lobe) of the lungs showed trends towards higher proinflammatory cytokine concentrations compared with the lower lobes (right lower lobe and left lower lobe). DISCUSSION/SIGNIFICANCE OF IMPACT: Our results demonstrate that P.a. variants (mucoid or nonmucoid) appear not to be geographically restricted in ability to colonize any lobe of the CF lung. Moreover, infection with mucoid P.a. (either alone or in mixed populations with nonmucoid variants) is associated with higher inflammatory cytokine concentrations in the CF lung. Given that infection with mucoid P.a. predicts deterioration in pulmonary function, this study provides a rationale for further investigation of cytokines as diagnostic/prognostic correlates of infection and lung disease in CF.

